# Characteristics of Participants Who Consented to Share Data with a Public Health Registry After an Environmental Disaster

**DOI:** 10.3390/ijerph22111630

**Published:** 2025-10-26

**Authors:** Marty Crawford, Diana K. Haggerty, Nicole Jones

**Affiliations:** 1 Michigan State University-Hurley Children’s Hospital Pediatric Public Health Initiative, Michigan State University, Flint, MI 48502, USA; hagger39@msu.edu (D.K.H.); warnerni@msu.edu (N.J.); 2 Charles Stewart Mott Department of Public Health, College of Human Medicine, Michigan State University, Flint, MI 48502, USA; 3 Department of Pediatrics and Human Development, College of Human Medicine, Michigan State University, Flint, MI 48502, USA

**Keywords:** consent to share data, tiered consent, public health intervention, disaster response

## Abstract

On 25 April 2014, the municipal water source in Flint, Michigan, was switched to the Flint River. Failure to properly treat the water for corrosion resulted in lead contamination of the water system. Resident concerns were dismissed by local, state, and federal government agencies until community participatory and clinical pediatric research demonstrated the contamination, and the water was restored to the original source 18 months later. Recovery efforts established a public health registry, funded by the Centers for Disease Control and Prevention. A grant of public health authority and registry funding were awarded to Michigan State University in August 2017 to establish a health surveillance system and public health intervention to refer exposed individuals to community services. Community feedback requested tiered data-sharing consent options. Participants who consented to join the registry were presented with four consent questions: to be contacted about future research opportunities, to use survey data to make referrals to services on their behalf, to share with the registry their State of Michigan Department of Health and Human Services (MDHHS) program data, and to share Michigan Medicaid administrative data. This descriptive study found that most participants consented to being contacted for future research (88%), sharing data for referrals (84%), and sharing data from MDHHS programs (77%) with the registry. Among participants with Medicaid insurance, 74% consented to sharing Medicaid data. Consent increased with age and decreased with income and education. Consent was higher among participants reporting food insecurity in the last 12 months. Consent to share data was unexpectedly high in the context of environmental disaster, trauma, and government distrust. Further work is needed to explore whether participation in public health activities is positively impacted by the implementation of a tiered consent process to share data.

## 1. Introduction

In April of 2014, the city of Flint experienced a human-caused environmental disaster that was the result of a cost-cutting effort directed by the city’s state-assigned emergency financial manager. The water source for the city of Flint was switched from pretreated water from the Detroit Water and Sewerage Department to water sourced from the Flint River and treated at the city of Flint water treatment plant. Local treatment processes used inadequate corrosion control for Flint River water, which caused corrosion of the protective pipe scale and subsequent leaching of lead into the water supply [1].

Resident concerns about water quality were repeatedly dismissed by local, state, and federal governments until community participatory and clinical pediatric research demonstrated the contamination [2,3]. It was 18 months before the contamination was acknowledged by the State of Michigan and a local lead-in-water emergency was declared, and 23 months before a federal emergency was declared [4]. Use of unfiltered tap water for drinking was common in the city of Flint [5], resulting in the exposure of more than 100,000 people to environmental contaminants, including lead [6]. Historical economic disinvestment in the city and disenfranchisement of Flint voters by state-appointed financial managers have resulted in community distrust of government agencies, which was further compounded by regulatory agencies’ disregard of community concerns during the Flint water crisis [7,8].

Ongoing monthly county-wide surveys noted an increase in perceptions of stress starting in October of 2015 when the lead emergency was announced, which continued through May of 2016; qualitative data identified both the water contamination and the sense of distrust in government officials as a frequently cited source of mental health distress [9]. Community surveys conducted in May 2016 found that 71.9% of residents felt stress due to being overlooked by decision makers, 31.2% reported only trusting themselves or did not trust anyone as a source of information about the Flint water crisis, and 10% said they had no trust in government [10]. In August 2016, just prior to the expiration of the federal emergency declaration, Flint residents reported a lower sense of trust than other Michiganders and other North Americans [11,12].

Amid this distrust, recovery efforts were launched to expand community, state, and federal resources to mitigate the impact of lead exposure [4]. In August 2017, the Centers for Disease Control and Prevention awarded funding to Michigan State University to establish a voluntary public health registry for persons impacted by the Flint water crisis between 24 April 2014 and 15 October 2015 [6]. In partnership with the community, the Flint Registry was launched in 2018 to improve health by conducting surveillance and connecting participants to existing programs and services via a coordinated service referral system [13].

Consent to share data was not required for joining the registry; however, it was an important part of the Flint Registry’s plan to share data with service providers for referrals and to access the State of Michigan’s public health data to follow long-term health needs and evaluate the impacts of Flint Registry participation. Aligned with requests from the community, the data-sharing consent question design used a tiered consent model to provide participants with a high degree of control over their data. Previous studies have often measured the hypothetical willingness to data sharing, i.e., one’s expected consent decision to share data for a hypothetical purpose in the absence of an actual study protocol and informed consent process, and often in the context of sharing medical or personal data for the purposes of research, rather than public health efforts. In general, high levels of hypothetical willingness to share data are reported [14,15,16,17]. Both the degree of data sensitivity and individual demographic characteristics such as gender, education, age, and income have been associated with differences in hypothetical willingness to share [15,18]. Finally, distrust or lower levels of trust have been correlated with reduced hypothetical willingness to share data for public health purposes [19,20]. However, it is unknown how levels of hypothetical willingness to share data might compare to consent rates, which could directly impact the effectiveness of interventions following environmental or public health crises.

In the context of prior research relative to hypothetical willingness to share data, the levels of distrust in the Flint community, which were exacerbated by the water crisis, and the association between distrust and hypothetical willingness to share data reported in the literature, we evaluated consent to share data with a health-promoting intervention implemented as part of a post-disaster response. The objectives of this study were to (1) describe the overall frequency of consent to share data with a public health project and (2) describe the sociodemographic characteristics associated with consent to share data following an environmental disaster. We anticipated that consent to share data would be low as compared to other studies reporting on hypothetical willingness to share and sought to further understand demographic differences in consent rates that could lead to bias in data collection for public health activities. Considering the dearth of studies in the literature evaluating consent to share data with public health registries, this study has the potential to shed light on whether such registries are able to achieve high data-sharing consent rates, specifically with a tiered consent process, as well as demonstrate the utility of evaluating consent rates among subpopulations, which could aid other public health interventions worldwide in designing their consent questions or identifying subgroups whose privacy concerns may require more attention.

## 2. Materials and Methods

Eligibility criteria to participate in the Flint Registry required only that an individual lived, worked, or attended school or day care in Flint during the water switch or was in utero during the period of the water switch. Surveillance is conducted through the administration of periodic Flint Registry surveys and access to select State of Michigan administrative data. The Flint Registry intervention supports participants’ connection to community resources for both immediate and long-term health, child development, nutrition, and lead mitigation and abatement needs [13]. Participants’ survey data are analyzed to identify individuals’ potential needs, and an electronic referral system provides a direct referral handoff from the Flint Registry to service providers. While Flint Registry enrollment was open to both children and adults, this paper focuses on adult participants’ consent to share data. Adult recruitment methods utilized both list-based mailings and outreach advertising and events. Invitation letters for recruitment were sent to individuals from a patient list from Hurley Medical Center and a list of individuals compiled from the Michigan Care Improvement Registry (MCIR) and Medicaid [6,13,21]. Broad community outreach efforts included radio and television advertising, bus wraps, and direct engagement at community events and public locations.

The Flint Registry enrollment process, which began in December 2018, included eligibility determination, consent to participate, optional consent to share data of varying sensitivity, and completion of a baseline survey. The FR enrollment process was offered in multiple modes of access (telephone interview, in-person interview, mailed paper-edition survey, and online survey) to reduce potential technology and literacy barriers, and interviewers completed training in relevant modules of NIH Good Clinical Practices for Social and Behavior Research [22] and received initial training in Response Sensitive Standardized Interviewing (RSSI, University of Wisconsin Survey Center, Madison, WI, USA) to support high-quality data collection and minimize interviewer bias and perceived coercion [13]. Independent of data-sharing consent decisions, a small appreciation check (USD 25–50) was mailed to participants who completed the baseline survey.

Consent to participate in (i.e., to join) the registry was obtained at the time of eligibility determination and followed by presentation of four additional consent questions: consent to use contact information for future contact about research opportunities (Q1 Contact); consent to share Flint Registry survey data with community resource providers for the purpose of making referrals (Q2 Referrals); consent to access Michigan Department of Health and Human Services (MDHHS) program data, including Women, Infants, and Children (WIC), MCIR, Vital Records, and Lead Safe Home, for the purposes of monitoring health and evaluating service utilization (Q3 Programs); and consent to share administrative, HIPAA-protected information from MDHHS Medicaid data for the purpose of monitoring health and evaluating service utilization (Q4 Medicaid) (Supplementary File S1).

Data-sharing consent questions Q1 Contact and Q2 Referrals were included at the start of registry enrollment in 2018. In 2021, a data use and non-disclosure agreement (DUA) between Michigan State University and MDHHS was executed, permitting the addition of data-sharing consent questions Q3 Programs and Q4 Medicaid. Unlike questions Q1 Contact, Q2 Referrals, and Q3 Programs, for which consent was collected simply as a verbal response to an interviewer’s reading of the question over the phone or by a participant’s digital response to the online survey consent question, Q4 Medicaid consent responses required the collection of participants’ signatures to satisfy the legal requirements to share Medicaid data. Signatures for Q4 Medicaid consent responses were collected as electronic (e-) signatures when completed as an online survey, or by a mailed exchange of paper forms between the Flint Registry and the participant when administered by an interviewer over the phone. Implementing e-signatures was a simple and effective modification to the original online consent process; however, collecting consent signatures by forms exchanged in the mail proved logistically infeasible. For this reason, the analysis of consent Q4 Medicaid includes records only for consent surveys completed online. For each of Q1–Q4, we considered a yes answer to be consent to share data for the circumstance presented in the question, so that an individual could consent to data sharing in one context but refuse to share in another.

We developed our descriptive analyses based on a framework for reporting descriptive epidemiologic studies [23]. Based on the prior literature, sociodemographic characteristics (race, ethnicity, income, education, health insurance, and economic stress) were selected to explore their associations with consent to share data. Participants chose all that applied from multiple race categories including White or Caucasian, Black or African American, Native American or Alaska Native, Asian, Native Hawaiian or Other Pacific Islander, Middle Eastern or North African, and Other, and responses were recoded as Black alone, White alone, Single race (not Black or White), and Multiracial to allow sufficient sample sizes. Ethnicity consisted of a single question asking participants if they were Hispanic, Latino/a, or of Spanish origin. Food insecurity was determined by asking, “Within the past 12 months, did the food you bought run out and you did not have any money to get more?” [24] and economic stress by asking, “If you lost all your current source(s) of household income (paycheck, public assistance, or other forms of income), how long could you continue to live at your current address and standard of living?” [25].

We used frequencies (percentages) to evaluate agreement and disagreement to participate in the Flint Registry and to consent to share data. Analyses were conducted using SAS 9.4 (SAS Institute, Cary, NC, USA). For this evaluation, participant counts of Flint Registry eligibility, participation, and consent to share data included all adults (18 years and older) surveyed between 1 December 2018 and 31 July 2022. Sociodemographic characteristic response frequencies of the adult cohort were determined for all those with a completed baseline survey, while data-sharing consent response frequencies were restricted to include only those who were presented with and answered the respective data-sharing consent questions (missing data excluded). Q4 Medicaid analysis included only those reporting current use of Medicaid insurance (those reporting no current use of Medicaid insurance were excluded). Chi-square goodness-of-fit tests were used to identify statistically significant bivariable associations. Due to the large sample size for Q1 and Q2, we used a more stringent alpha, and *p*-values of 0.01 were considered statistically significant. For Q3 and Q4, we used *p* < 0.05 to identify statistically significant associations. To help distinguish important patterns further for Q1 and Q2, we highlighted bivariable frequency differences of 5% in the results.

## 3. Results

**Figure 1:**
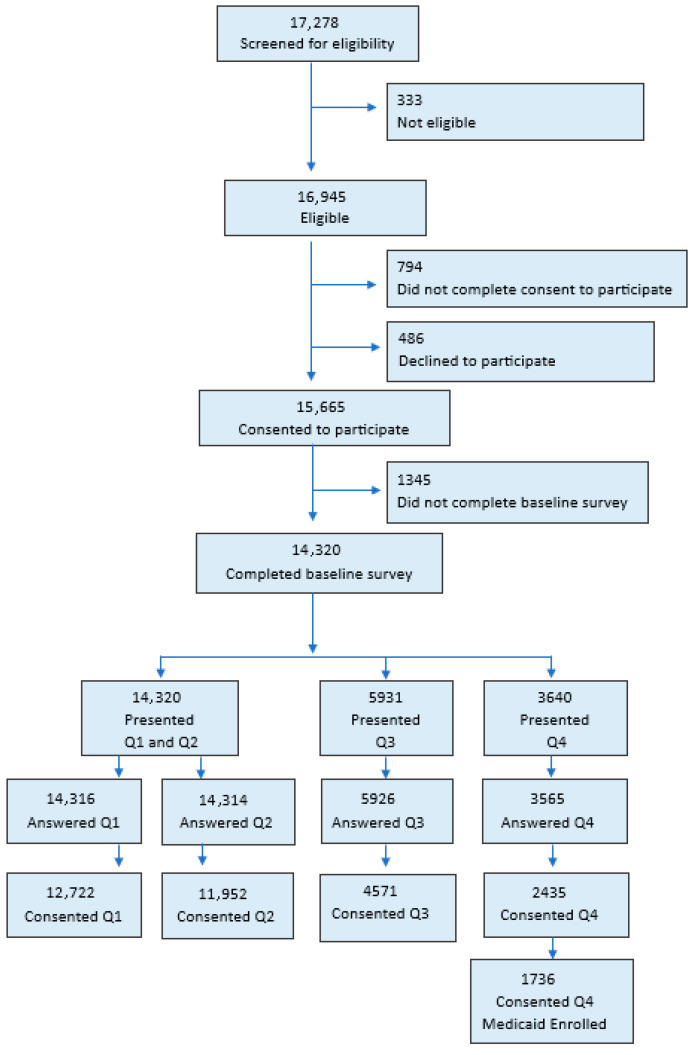
Counts of (potential) participants from Flint Registry eligibility screening through consenting to share data.

Of those individuals who expressed interest in the Flint Registry and completed eligibility screening (n = 17,278), 98.1% (n = 16,945) were determined to be eligible to participate in the Flint Registry, 90.7% (n = 15,664) consented to participate in the Flint Registry, and 91.4% (n = 14,320) of those who consented completed the baseline survey (Figure 1). Data-sharing questions Q1 Contact and Q2 Referrals were completed by nearly all participants who completed the baseline survey (99.9%, n = 14,316, and 99.9%, n = 14,314, respectively). Consent questions Q3 Programs and Q4 Medicaid, added to the consent process nearly 2 years after the start of enrollment, were presented to 5931 and 3640 participants and were also answered by nearly 100% of participants who were asked (99.9%, n = 5926, and 97.9%, n = 3565, respectively) (Figure 1).

Sociodemographic characteristic frequencies, including age, race, ethnicity, gender, education, income, health insurance, food insecurity, and others, were determined for participants with complete baseline surveys (Table S1). The cohort comprised more women than men (68.5% versus 31.3%) and more individuals of Black only race than those of White only (60.0% versus 31.8%).

Most were neither Hispanic nor Latino (95.9%), and more than half were currently unmarried (68.9%, n = 9785). More than half of the participants reported an annual income of USD 25,000 or less (57.0%, n = 8067) and earning a high school diploma (32.6%, n = 4637) or completing some college (38%, n = 5401). One-third said in the last 12 months, they had run out of food before they had money to buy more (33.2%, n = 4724).

A majority of participants consented to share data with the Flint Registry, but the proportion decreased as the sensitivity of the data to be shared increased (Table 1). Participant consent was greatest to share contact data for outreach regarding future research opportunities (Q1 Contact: 88.8%, n = 12,722) and least to share MDHHS Medicaid administrative data for linking to Flint Registry survey data (Table 1). Q4 Medicaid consent (n = 1275) for all adults was 66.9% (N = 3640) and 73.4% (N = 1710) for only adults enrolled in Medicaid at the time of survey administration.

Participants’ agreement to consent to future contact and data sharing for referral purposes increased as age increased (*p* < 0.0001) (Table 2). A threshold effect of income for Q1 Contact was noted at USD 35,000, whereas it was noted at USD 25,000 for Q2 Referrals. Participants who reported a bachelor’s degree or higher were less likely to agree to sharing their data to receive referrals (77.9% versus 83.1–86.2%, *p* < 0.0001). This pattern was not reflected in Q1 Contact. Participants who reported running out of food without money to buy more were more likely to respond yes to sharing data for referrals (88.6%) and for future contact (92.0%) compared to those who did not run out of food (81.1% and 87.5%, respectively; *p* < 0.0001 for both).

Consent to allow the Flint Registry to access MDHHS program data and Medicaid administrative data increased as age increased, though this was statistically significant for Q3 MDHHS sharing (*p* < 0.0001) but not for Q4 Medicaid data (Table 3). Black participants and participants who reported their race as neither White nor Black, or who reported their race as multiracial, less frequently agreed to share their Medicaid data with the Flint Registry compared to White participants, though this was not significant. Consent to share Medicaid data varied by income, but the pattern was not consistent; participants whose annual household income was between USD 25,000 and USD 34,999 and above USD 50,000 were less likely to consent to sharing Medicaid data (*p* < 0.05). Participants who reported running out of food in the last 12 months and did not have enough money to buy more consented more frequently to Q3 Programs and Q4 Medicaid (Q3: 81.6%, Q4: 78.3%) than those who did not run out of food in the last 12 months (Q3: 75.5%, Q4: 72.3%, *p* < 0.01 for both questions).

## 4. Discussion

The city of Flint, Michigan, experienced an environmental disaster caused by government error that started in April 2014 and continued unacknowledged for a period of 18 months. In implementing this public health project designed to support post-disaster recovery, we considered that higher levels of community distrust, existing prior to and further exacerbated by the water crisis (see below), would limit participant consent to share data for the purposes of health promotion referrals, health outcome surveillance, and program evaluation. However, most participants consented to share data both with our public health registry and indirectly with community programs for the purpose of referrals to support nutrition, healthcare, and lead abatement (range: 73.4% to 88.8%). Our consent process was developed in collaboration with the community, who requested a tiered data-sharing model to provide participants with a high degree of control over their data [26]. Four consent questions related to types of data to be shared were presented, and those for the most sensitive data, MDHHS program data (Q3) and Medicaid administrative data (Q4), had the lowest proportions of participant consent to share. Sharing data for the purpose of making participant referrals (Q2), which offers a potential personal benefit of being connected to service providers, had 83.5% of participants’ consent. Participants with higher income and education levels were less likely to consent to share these data (Q1 and Q2) as compared to participants with lower incomes and education levels. Other sociodemographic characteristics, such as ethnicity, gender, and health insurance status, did not relate to consent to share data.

Just prior to the expiration of the federal emergency declaration, Flint residents reported a lower sense of trust than other Michiganders and other North Americans [11,12]. A cross-sectional study conducted between June 2016 and October 2017 asked Flint residents about trust in elected officials, bureaucrats, researchers, external organizations, and nonprofits before and after the water crisis. They reported a significant decrease in trust overall after the water crisis, and that residents were less trusting of governmental than non-governmental organizations; a greater decrease in trust with the government was observed as well [27]. Further, in July 2018, 57% of residents reported experiencing stress due to being overlooked by decision makers due to the water crisis, indicating the negative impact on trust in the community was likely persistent [28]. The economic disinvestment in Flint that started in the 1980s and the subsequent financial challenges of the city of Flint were documented prior to the start of the water crisis, and community members expressed skepticism that efforts of government agencies to fix the damage of the water crisis were made in good faith, citing these historical precedents and beliefs that agencies knew what was happening but failed to protect the Flint community [7,8].

We considered that this low community trust would result in a lower frequency of participants consenting to share data as compared to other studies. However, we found the proportion to be comparable and even higher in comparison to some previous work on hypothetical willingness to share medical data for research or public health purposes. Prior work in the United States looking at hypothetical willingness (i.e., one’s expected consent decision to a proposed (i.e., simulated) consent question and consent process) to share medical data for research has found ranges of 62.4–91.3% among patients who were asked to share data about their children, 50–90% among a group of faculty, staff and students from a medical center, approximately 45% in patients seeking care in a medical department, and 57.7–61.5% in an online sample of people with medical conditions [15,17,29,30].

Like our work reporting actual consent decisions, these studies of hypothetical consent found that the type of data asked for influenced the hypothetical willingness to share. Contrary to our finding for data sharing of medically related information found in Medicaid, a large research registry of volunteers for recruitment to future studies found much higher rates of data sharing for medical record data (97.7%) than our project [18], which is possibly a result of participant self-selection to participate. Finally, previous findings relative to demographic differences and hypothetical willingness to share data have been mixed [31]. In comparison to other studies looking at medical record sharing, we identified demographic differences such as education and income that have been previously reported, but we did not see age and gender differences [15,18]. We identified a new association between greater consent to share data and those experiencing food insecurity within the past 12 months as compared to those who did not.

In the case of the Flint Registry, a non-governmental entity implemented a public health response. It is possible that leadership by Michigan State University as a non-governmental agency and the context of the public health emergency increased data-sharing consent despite persistent distrust in the community. A prior study found that patients are more willing to share medical record data for research in a public health emergency and that using a trusted intermediary would increase their hypothetical willingness to share [14]. Further, an online survey of a random sample found that slightly more people said they were confident that a university hospital would protect their health information versus a local public health department [32]. In a Canadian survey, people reported that they were much more likely to trust university researchers versus local government to keep health information confidential [33]. Michigan State University built upon existing, local relationships and expanded partnerships as part of the Flint Registry implementation and invested time into a broad community engagement process [21]. We suggest that the community outreach strategy, which was central to recruitment, may have increased consent to share data. Over 50% of enrollees heard about the Flint Registry through a community-engaged recruitment method such as public presentation, a community organization, word of mouth, or a community ambassador [21].

The strengths of this work are its large, diverse sample and city-wide implementation of a public health intervention. Due to the time lag in receiving funding for the project and the time spent engaging with the community prior to launching the project, data were collected 4–8 years after switching back to water sourced from the city of Detroit. It is unknown whether this influenced consent to share data. In our case, government decisions and inadequate oversight of the water treatment system led to the crisis. These factors mean our results may be less generalizable to other incidents of environmental disaster. These results are applicable to other public health promotion interventions that require data sharing for evaluation and implementation. The demographic make-up of the Flint Registry differs from the composition of the city of Flint. Our sample includes residents of the city of Flint and people who live outside the city because of the broad inclusion criteria of the Flint Registry. Our sample has a higher proportion of adult female participants than expected (approximately 68%), which may limit the generalizability to other populations. The Flint Registry also has a higher proportion of Black/African American participants than is found in the United States Census estimates for the city of Flint [34].

Further research is needed to understand specific concerns related to data sharing for public health purposes. However, relative to sharing data for research, lack of trust that information would remain anonymous, lack of trust that information would be treated as sensitive, possible discrimination, other confidentiality concerns, concerns about effects on medical care, and worries about health insurance issues have been listed as reasons for not sharing [14,17,29]. While we did not specifically collect data on reasons for refusing to share data, at community events and town halls, concerns about data privacy were the most common question.

## 5. Conclusions

### Public Health Implications

Our community made it clear that control over data sharing and privacy were critical considerations for the Flint Registry to address. The Flint Registry used tiered consent for data sharing to address these concerns, and participants, by and large, agreed to have their data shared with community partner organizations and other research studies, and to share MDHHS program and Medicaid administrative data with the Flint Registry. Our experience supports the use of tiered consent questions that allow participants to control the type of and degree to which their data are shared with partner organizations and the extent to which other organizations may share their data with the organization that is conducting surveillance efforts. Our findings suggest that tiered consent supports participant autonomy and may drive consent decisions. However, additional research is needed to understand its generalizability on consent rates and to determine if tiered consent specifically impacts participant trust in surveillance systems. Identifying subpopulations within a study that consent less frequently to share data will enhance reporting of data limitations and may point to data privacy needs that require alternative solutions. Particularly for public health surveillance systems with open enrollment, assessing data-sharing consent patterns can identify subgroups whose concerns need additional attention.

Our results suggest that post-disaster public health interventions can successfully capture a high level of consent to share data. Community engagement strategies and tiered consent questions to share data may increase consent to share. Further research is needed to understand concerns relative to data sharing post-disaster and to develop best practices for consent processes to address demographic differences in public health activities in the United States and internationally.

## Figures and Tables

**Table 1:** Participant responses to data-sharing consent questions.

	Q1 Contact N = 14,316 % (n)	Q2 Referrals N = 14,316 % (n)	Q3 Programs N = 5926 % (n)	Q4 Medicaid ^a^ N = 1736 % (n)
Yes	88.8% (12,722)	83.5% (11,952)	77.1% (4571)	73.4 (1275)
No	11.1% (1594)	16.5% (2362)	22.8% (1355)	25.1 (435)

Missingness ranged from 0.03 to 1.5%. ^a^ Q4 Medicaid includes only those reporting “currently enrolled” in Medicaid at the time of survey administration.

**Table 2:** Bivariable associations of sociodemographic characteristics of Flint Registry participants with consent to share contact information for future studies and consent to share contact information for referrals.

	Q1 Contact N Yes = 12,722 N Total = 14,316	Q2 Referrals N Yes = 11,952 N total = 14,316
Characteristics	% (N)	% (N)
Age Categories		
18–24 years	83.4 (1068) ***	76.9 (984) ***
25–39 years	86.4 (3550) ***	80.2 (3296) ***
40–65 years	90.7 (6134) ***	85.4 (5780) ***
>65 years	91.3 (1970) ***	87.6 (1892) ***
Race		
White	86.3 (3893) ***	81.0 (3654) ***
Black	90.1 (7679) ***	84.9 (7229) ***
Single race, neither White nor Black	87.9 (312) ***	82.8 (293) ***
Multiple races	90.3 (638) ***	84.0 (564) ***
Don’t know and Missing	200	182
Ethnicity		
Hispanic/Latino/a or Spanish	90.1 (455)	86.7 (438)
Non-H/L/S	88.8 (12,117)	83.4 (11,377)
Don’t know and Missing	150	137
Gender		
Female	89.0 (8706)	83.7 (8185)
Male	88.5 (3951)	83.1 (3710)
Other	94.1 (16)	76.5 (13)
Don’t know and Missing	49	44
Income		
<$25,000	90.5 (7299) ***	85.8 (6919) ***
$25,000–$34,999	88.8 (1421) ***	83.8 (1342) ***
$35,000–$49,999	85.3 (1261) ***	83.0 (1195) ***
$50,000–$74,999	85.3 (935) ***	78.6 (861) ***
$75,000+	84.4 (763) ***	77.0 (696) ***
Don’t know and Missing	1043	939
Education		
Less than HS Diploma	90.2 (1526) **	86.2 (1458) ***
HS Diploma/GED	88.3 (4095) **	83.1 (3850) ***
Some College, Associate’s Degree, or Technical School (college 1–3 years)	89.8 (4846) **	85.6 (4618) ***
Bachelor’s, Master’s, or Professional Degree	86.9 (2124) **	77.9 (1904) ***
Don’t know and Missing	131	122
Health Insurance		
Yes	81.9 (11,662)	83.6 (10,948)
No	88.1 (787)	84.7 (756)
Don’t know and Missing	273	248
If you lost current sources(s) of income, how long could you continue to live at your current address and standard of living?		
1–2 months	89.5 (3629)	85.5 (3464) **
3–6 months	90.6 (2860)	85.8 (2707) **
7–12 months	88.7 (1309)	82.9 (1223) **
12–18 months	90.2 (1416)	82.8 (1300) **
Don’t know and Missing	3508	3258
Within past 12 months, did the food you bought run out and you did not have any money to get more?		
Yes	92.0 (4344) ***	88.6 (4184) ***
No	87.5 (8078) ***	81.1 (7488) ***
Don’t know and Missing	300	280
Medicaid, Currently enrolled		
Yes	90.8 (6119) ***	85.9 (5789) ***
No	87.8 (2909) ***	81.6 (2706) ***
Don’t know and Missing	3694	3457

*** Chi-square goodness-of-fit *p*-value < 0.0001. ** Chi-square goodness-of-fit *p*-value < 0.01. Total denominator for each variable includes no responses to consent questions.

**Table 3:** Bivariable associations of sociodemographic characteristics of Flint Registry participants with consent to share Michigan Department of Health and Human Services program data and Medicaid administrative data with the Flint Registry.

	Q3 Programs N Yes = 4571 N Total = 5926	Q4 Medicaid ^a^ N Yes = 1275 N total = 1736
Characteristics	% (N)	% (N)
Age Categories		
18–24 years	71.3 (397) ***	71.9 (146)
25–39 years	74.2 (1301) ***	73.4 (522)
40–65 years	78.9 (2125) ***	76.2 (563)
>65 years	81.1 (748) ***	77.2 (44)
Race		
White	78.0 (1425)	77.6 (485)
Black	76.8 (2714)	73.3 (666)
Single race, neither White nor Black	79.3 (126)	68.1 (32)
Multiple races	77.3 (223)	71.0 (66)
Don’t know and Missing	83	26
Ethnicity		
Hispanic/Latino/a or Spanish	76.3 (190)	71.0 (61)
Non-H/L/S	77.2 (4338)	74.8 (1203)
Don’t know and Missing	43	11
Gender		
Female	76.7 (2950)	73.3 (891)
Male	78.0 (1590)	77.7 (377)
Other	85.7 (6)	0
Don’t know and Missing	25	7
Income		
<$25,000	78.8 (2540) *	77.0 (970) *
$25,000–$34,999	77.7 (544) *	70.5 (129) *
$35,000–$49,999	74.7 (438) *	77.5 (62) *
$50,000–$74,999	74.0 (327) *	60.9 (28) *
$75,000+	74.8 (231) *	66.7 (6) *
Don’t know and Missing	491	80
Education		
Less than HS Diploma	80.6 (607)	78.4 (185)
HS Diploma/GED	76.9 (1647)	73.1 (476)
Some College, Associate’s Degree, or Technical School (college 1–3 years)	77.1 (1628)	74.3 (505)
Bachelor’s, Master’s, or Professional Degree	75.2 (640)	77.6 (97)
Don’t know and Missing	49	12
Health Insurance		
Yes	77.6 (4177)	74.6 (1275)
No	76.4 (288)	0
Don’t know and Missing	106	0
If you lost current sources(s) of income, how long could you continue to live at your current address and standard of living?		
1–2 months	77.4 (1262)	76.1 (473)
3–6 months	78.1 (987)	73.4 (259)
7–12 months	78.5 (522)	85.4 (88)
12–18 months	81.3 (598)	72.5 (71)
Don’t know and Missing	1202	384
Within past 12 months, did the food you bought run out and you did not have any money to get more?		
Yes	81.6 (1404) ***	78.3 (511) **
No	75.5 (3058) ***	72.3 (730) **
Don’t know and Missing	109	34
Medicaid, Currently enrolled		
Yes	79.6 (2284) **	74.6 (1275)
No	76.0 (1114) **	0
Don’t know and Missing	1173	0

*** Chi-square goodness-of-fit *p*-value < 0.0001. ** Chi-square goodness-of-fit *p*-value < 0.01. * Chi-square goodness-of-fit *p*-value < 0.05. ^a^ Q4 Medicaid included only participants reporting “currently enrolled in Medicaid” at the time of survey completion. Total denominator for each variable includes no responses to consent questions.

## Data Availability

The raw data supporting the conclusions of this article will be made available by the authors on request. Requested data may be provided after IRB approval and appropriate data use agreements have been obtained.

## Supplementary Materials

The following supporting information can be downloaded at https://www.mdpi.com/article/10.3390/ijerph22111630/s1, File S1: Flint Registry adult consent form; Table S1: Sociodemographic characteristics of Flint Registry participants (Total N = 14,320).
