# Supplementary material for: Characteristics of Participants Who Consented to Share Data with a Public Health Registry After an Environmental Disaster
Source: Int J Environ Res Public Health. 2025 Oct 26;22(11):1630. doi: 10.3390/ijerph22111630 (PMC12652031; doi:10.3390/ijerph22111630)
Supplement: Supplementary file 1 [file ijerph-22-01630-s001.zip › Supplementary Material_Flint Registry Adult Consent Form.pdf]

Flint Registry/Adult Consent Form

This is the consent version that can be completed and signed in-person.

Adult consent to enroll

The next section asks about your decision to participate in the Registry and is called the consent. The consent section must be completed by the adult who is enrolling in the Registry or by the adult's official legal representative.

Are you completing this on behalf of someone else?

☐ Yes

☐ No

If Yes, what is your relationship to the adult who is enrolling in the Flint Registry:

☐ I am currently his/her Durable Power of Attorney

☐ I am currently his/her Legal Guardian

About the Flint Registry

The goals of the Registry are to connect people to services, to document the health effects of the Flint water crisis, and to promote wellness and recovery. The Flint Registry is for adults and children exposed to the Flint Water System from April 25, 2014 - October 15, 2015.

The Registry is sponsored by the Centers for Disease Control and Prevention and led by a Flint-based team at Michigan State University (MSU). MSU works with many groups in the community including the City of Flint, Greater Flint Health Coalition, educators, clinicians, community-based organizations, and most importantly, residents of Flint, to make sure the Registry reflects the needs of the community.

Participating in the Flint Registry is voluntary. If you decide not to enroll in the Registry, you will not lose any rights or benefits that you would otherwise get. Please take your time to make your choice about participating. If you have questions, at any time, please contact Flint Registry staff by phone at 833-GO-FLINT or email at flintregistry@hc.msu.edu.

Here are some frequently asked questions about the Flint Registry. More information is also available at FlintRegistry.org

This is the consent version that can be completed and signed in-person.

What will happen if I join the Flint Registry?

✓ **Step 1-Complete your Survey**

If you choose to enroll, you will be asked to fill out a survey. This survey will include questions about your background, health, and exposure to Flint water. You can choose one of these ways to complete your survey: online, over the phone, at the Flint Registry office, or by filling out and mailing back a paper copy of the survey. After completing the survey, you will be mailed a thank you check.

✓ **Step 2-Get Referred**

If you agree to be referred to services, the Flint Registry team will share your information and connect you to community services you may be eligible for. These services may include medical insurance, nutrition support, and home lead identification and fixing.

✓ **Step 3 - Complete your follow-up survey**

About one year after completing your first survey, you will be asked to fill out a follow-up survey**.**

✓ **Step 4 - Future Surveys**

The Registry may continue to be funded and to contact you about future surveys.

Can I stop participating in the Registry if I change my mind?

Yes. You can decide to leave the Registry at any time.

How does the Registry use, protect, and share information?

Any information gathered by the Registry about you will be kept confidential. Information that identifies you will not be shared without your consent unless we are required by law to disclose information.

In rare cases, we may be required to reveal confidential information related to safety. We are required to report suspected cases of child abuse, or if you tell us you are planning to cause serious harm to yourself or others, or if we reasonably believe you are a threat to yourself or others.

This is the consent version that can be completed and signed in-person.

Information will be kept in a secure database at MSU and Hurley Medical Center. The databases will only be available to Registry staff and the Registry's contractors.

We will do our best to make sure that the personal information gathered for this study is kept private. We have taken multiple steps to protect your information by using password protected systems with physically secured servers. All Flint Registry workers are required to receive training in ways to keep information secure.

Information from the Registry will be made available to Flint residents and community members in the form of group reports. Your name will not be connected with your answers. Information may be shared with researchers and be published or presented at professional meetings, but there will be no information that identifies you, like your name or your birthdate. Unless you agree, your name and contact information will not be shared with anyone.

What are the benefits of participating in the Registry?

You may personally benefit from your participation if Registry staff are able to refer you to services. Other people may benefit because information collected in the Flint Registry may help us understand how lead exposure affects health.

What are the possible risks of participating?

The possible risks of participating are that some of the survey questions may remind you of your feelings or issues you faced during the water crisis. The Registry will provide information on community resources to help with your feelings.

If you have questions, please contact us at FlintRegistry@hc.msu.edu or call 833-GO-FLINT.

Join the Registry

Based on the information provided, would you like to join the Registry?

☐ Yes. Please enroll me in the Flint Registry.

☐ No. Do NOT enroll me in the Flint Registry.

This is the consent version that can be completed and signed in-person.

There are additional decisions to make about participating in the Registry:

Decision 1: Let the Registry help you access resources

The Registry's survey will ask questions about your health, household information, and exposure to lead. Using your answers, the Registry will help identify the services in the community that you may be eligible for.

Do you choose to "give permission" or to "NOT give permission" for the Registry to share your survey information with service providers to help enroll you in programs and services?

- ☐ Yes. I give my permission for the Flint Registry to share my information with service providers.
- ☐ No. I do NOT give my permission for the Flint Registry to share my information with service providers.

Decision 2: Allow the Flint Registry to contact you about other projects

Research on the water crisis is important to further our understanding of how to help children and adults exposed to lead and similar crises. Do you choose to "give permission" or to "NOT give permission" for the Registry to contact you in the future to learn about other projects?

- ☐ **Yes.** I give my permission for the Flint Registry to contact me in the future about participating in other projects.
- ☐ **No.** I do NOT give my permission for the Flint Registry to contact me about participating in other projects.

MDHHS Consent Decision: Allow the Michigan Department of Health and Human Services (MDHHS) to provide some of your program information to the Flint Registry

The Registry would like to obtain information about your health and well-being from your MDHHS records for the following programs: Michigan Care Improvement Registry (MCIR), Childhood Lead Poisoning Prevention Program (CLPPP), Vital Records, Women Infants and Children (WIC), and Lead Safe Home. **Giving permission to MDHHS to share your information is voluntary and refusing does not affect your eligibility for any MDHHS programs or services.**

This is the consent version that can be completed and signed in-person.

With your permission, we can link this information with other information in the Registry to help us to better understand the impact of lead-exposure on your health and to understand how using services from the State of Michigan may have affected your health. Your permission to allow MDHHS to provide some of your information to the Flint Registry ends when the Registry ends, unless you choose to withdraw your permission in writing before that time.

☐ **Yes.** I give my permission for the MDHHS to provide my information from MCIR, CLPPP, Vital Records, WIC, and Lead Safe Home to the Flint Registry.

☐ **No.** I do NOT give my permission for the MDHHS to provide my information from MCIR, CLPPP, Vital Records, WIC, and Lead Safe Home to the Flint Registry.

This is the consent version that can be completed and signed in-person.

Medicaid Consent Decision: Allow the Michigan Department of Health and Human Services (MDHHS) to provide some of your HIPAA-protected health information to the Flint Registry

The Registry would like to obtain information about your health and well-being from your Medicaid records held by the MDHHS. Medicaid records are governed by the Health Information Portability and Accountability Act (HIPAA). **Giving permission to MDHHS to share your Medicaid information is voluntary and refusing does not affect your eligibility for any MDHHS programs or services.**

With your permission, we can link this information with other information in the Registry to help us to better understand the impact of lead-exposure on your health and to understand how using Medicaid services from the State of Michigan may have affected your health. Your permission to allow MDHHS to provide some of your Medicaid information to the Flint Registry ends when the Registry ends, unless you choose to withdraw your permission in writing before that time. Any information gathered by the Registry about you will be kept confidential. Information that identifies you will not be shared without your consent unless we are required by law to disclose information. If we are required by law to disclose information, it is possible that this information will no longer be protected under HIPAA.

You may request a copy of your decision, or you may change your decision to share data at any time by submitting a Request to Withdraw form. The form may be found at FlintRegistry.org, or you may ask for a copy to be mailed by calling the Registry at 833-GO-FLINT (833-463-5468).

☐ **Yes.** I give my permission for the MDHHS to provide my Medicaid information to the Flint Registry.

☐ **No.** I do NOT give my permission for the MDHHS to provide my Medicaid information to the Flint Registry.

What is your relationship to the person enrolling in the Flint Registry: (check one)?

☐ Self

☐ I am currently his/her Durable Power of Attorney

☐ I am currently his/her Legal Guardian and am responsible to act on behalf of him/her in matters of care or custody.

Flint Registry/Adult Consent Form

This is the consent version that can be completed and signed in-person.

Participant information and signature

Please print information below:

____

First Name Middle Initial Last Name

____

Date of Birth

____

Address

____

City State Zip Code

Signature ____Date______________________
