# Supplementary material for: Characteristics of Participants Who Consented to Share Data with a Public Health Registry After an Environmental Disaster
Source: Int J Environ Res Public Health. 2025 Oct 26;22(11):1630. doi: 10.3390/ijerph22111630 (PMC12652031; doi:10.3390/ijerph22111630)
Supplement: Supplementary file 1 [file ijerph-22-01630-s001.zip › Supplementary Material_Table S1.pdf]

Supplementary Material

Table S1. Sociodemographic characteristics of Flint Registry participants (Total N=14,320).

Characteristic	% (N)
**Age**	
18–24 years	9.0 (1,282)
25–39 years	28.7 (4,111)
40–65 years	47.3 (6,768)
> 65 years	15.1 (2,159)
**Race**	
White	31.8 (4,505)
Black	60.0 (8,508)
Single race, neither White nor Black	2.5 (348)
Multiple races	5.2 (732)
Don’t know	0.5 (76)
Missing	151
**Ethnicity**	
Hispanic/Latino/a or Spanish	3.6 (505)
Non-H/L/S	95.9 (13,645)
Don’t know	0.5 (72)
Missing	98
**Gender**	
Female	68.5 (9,783)
Male	31.3 (4,465)
Other	0.1 (17)
Don’t know	0.1 (10)
Missing	45
**Currently Married**	
Yes	30.3 (4,306)
No	68.9 (9,785)
Don’t know	0.8 (117)
Missing	112
**Income**	
< $25,000	57.0 (8,067)
$25,000–$34,999	11.3 (1,601)
$35,000–$49,999	10.2 (1,440)
$50,000–$74,999	7.7 (1,096)
$75,000+	6.4 (904)
Don’t know	7.4 (1,048)
Missing	164
**Education**	
Less than HS Diploma	11.9 (1,691)
HS Diploma/GED	32.6 (4,637)
Some College, Associate’s Degree, or Technical School (college 1–3 years)	38.0 (5,401)
Bachelor’s, Master’s, or Professional Degree	17.2 (2,445)
Don't know	0.3 (47)
Missing	99

**Currently have health insurance**	
Yes	91.9 (13,094)
No	6.3 (893)
Don't know	1.9 (267)
Missing	66
**If you lost current source(s) of income, how long could you continue to live at your current address and standard of living?**	
1–2 months	28.9 (4,055)
3–6 months	22.5 (3,157)
7–12 months	10.5 (1,476)
12–18 months	11.2 (1,571)
Don't know	27.0 (3,796)
Missing	265
**Within the past 12 months, did the food you bought run out and you did you not have any money to get more?**	
Yes	33.2 (4,724)
No	64.9 (9,234)
Don't know	1.9 (265)
Missing	97
**Currently enrolled in Medicaid**	
Yes	65.1 (6,746)
No	32.0 (3,315)
Don't know	2.9 (297)
Missing	3962
